# Structural behaviour and construction of a monumental ice structure

**DOI:** 10.1016/j.heliyon.2024.e37312

**Published:** 2024-09-02

**Authors:** Arno Pronk, Elke Mergny, Qingpeng Li, Yiling Zhou

**Affiliations:** a Department Built Environment, Eindhoven University of Technology, 5600, MB Eindhoven, the Netherlands; b School of Architecture, Tianjin University, Tianjin, 300072, China; c Canopy Institute of Design, Beijing, 100026, China

**Keywords:** Ice shell, Pykrete, FRP mould, Ice composite, Renewable, Bio based, Steel frame

## Abstract

This paper investigates the use of ice-based materials combined with Fiber-Reinforced Polymer (FRP) to enhance the bending capacity of ice sculptures. Through three-point bending tests and ABAQUS simulations, the study analyses the mechanical behaviour of GFRP and cellulose ice, both separately and together. The results show that FRP improves tensile resistance while cellulose ice adds mass and ductility. An ABAQUS simulation with a layered shell partially captures the mechanical behaviour but has limitations in modelling beyond the elastic phase due to assumptions of perfect connection and material failure. Applied to a monumental sculpture by artist KAWS, incorporating a steel framework, GFRP mould, and ice-based material, the study confirms the potential of combining FRP and cellulose ice to achieve improved structural performance in ice-based sculptures.

## 1 Introduction

**Fig. 1:**
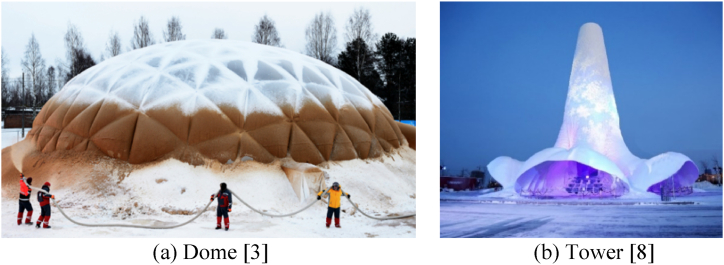
Ice shell structures.

Ice is a popular medium for sculpture. Artists traditionally carve ice blocks, assembling them much like they would in constructing traditional brick masonry structures. These “ice masonry” structures have the advantage of easy construction, but their shape, span and height are limited. Over the past decade, a new wave of techniques has emerged in the construction of ice structures. These methods involve projecting ice-based materials onto an inflatable fabric formwork, where the shape is adjusted with the use of ropes. The ice gradually freezes layer by layer to form a solid ice shell (Fig. 1). Initially pioneered by Isler [1] and further refined by Kokawa [2], Pronk [[3], [4], [5]], Coar [6,7], and others, this inflatable-formwork construction method draws inspiration from thin-shell concrete structures developed by Californian architect Wallace. This method makes it easier to build shell structures such as domes and also enables the construction of towers [8].

**Fig. 2:**
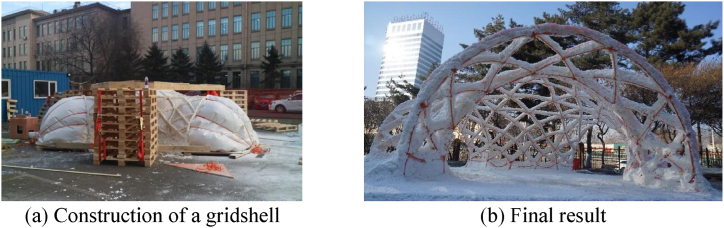
3D Ice gridshell structure [9].

**Fig. 3:**
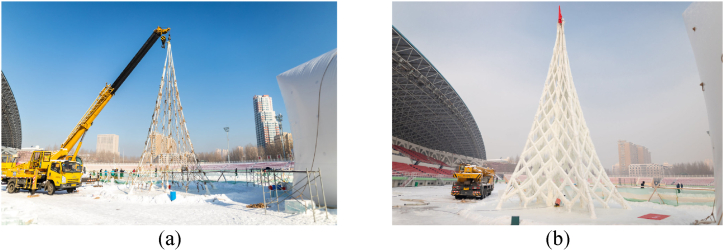
Truss tower in Harbin (2019) [10].

Ice is not limited to forming shells; it can also be used to create grid shells [9]. The same process described earlier can be applied, but instead of projecting ice-based materials onto an inflatable fabric formwork, layers of ice are applied only to the ropes (Fig. 2). Another notable variation of this process entails spreading the material onto a network of ropes suspended by a crane. A compelling example of this innovative method was showcased in the construction of a tower in Harbin in 2019 (Fig. 3) [10]. These advancements have expanded the possibilities for creating complex and architecturally impressive structures.

Beyond advancing construction techniques for ice sculptures and structures, significant improvements have been made in the ice material itself. Utilizing plain ice as a construction material indeed poses significant challenges due to its inherently weak structure, high temperature sensitivity, and unpredictable mechanical properties. To mitigate these issues, in 1942, Pyke developed ‘Pykrete’ [11], a novel composite material blending ice with sawdust. Following this innovation, Nixon [12] conducted research to assess the fracture toughness and bending strength of various ice composite materials. Further studies by Cruz and Belis [13] focused on the compressive strength of ice-cellulose composites. In a significant improvement, Li et al. [14] developed Ultimate Pykrete that has properties of high strength and low weight.

However, ice, even with additives, is primarily used in structures where compression dominates. Bending-based frames are less common due to the material's low tensile strength. Adding reinforcement, such as Fiber-Reinforced Polymer (FRP), can be beneficial. FRP is a composite material comprising a polymer matrix reinforced with fibres, commonly glass, carbon, aramid, or basalt, with occasional use of other fibres like paper or wood. The polymer matrix typically consists of epoxy, vinyl ester, or polyester thermosetting plastic, with some applications still using phenol-formaldehyde resins. This material is used in aerospace, military, naval, and automotive applications. The use of FRP and ice is discussed in the article by Coar et al. [15], where a complex shape is created by combining ice with FRP rebars.

This paper explores how an ice-based material and FRP can be combined to improve bending capacity through three-point bending tests. The principle is applied to a sculpture designed by the American contemporary artist KAWS. The sculpture is constructed by projecting a special ice-based material (called cellulose ice) onto a mould made of Glass Fiber-Reinforced Polymer (GFRP), which is supported by a steel structure. To ensure a strong connection between the cellulose ice and GFRP, the sculpture is also covered with fabric. The study uses an ABAQUS model to confirm the strength of the ice-GFRP-steel frame system.

## 2 Ice-based material and Glass Fiber-Reinforced Polymer

Inspired by Coar et al. [15], tests were conducted to evaluate the potential of this material combination. In this section, three-point bending tests are performed on GFRP and cellulose ice beams, both separately and together.

The objective is to compare the mechanical behaviour of the composite material with that of the individual components. Subsequently, a numerical model is established in ABAQUS to simulate the mechanical behaviour of the composite specimen.

### 2.1 3-Point bending test on GFRP

**Fig. 4:**
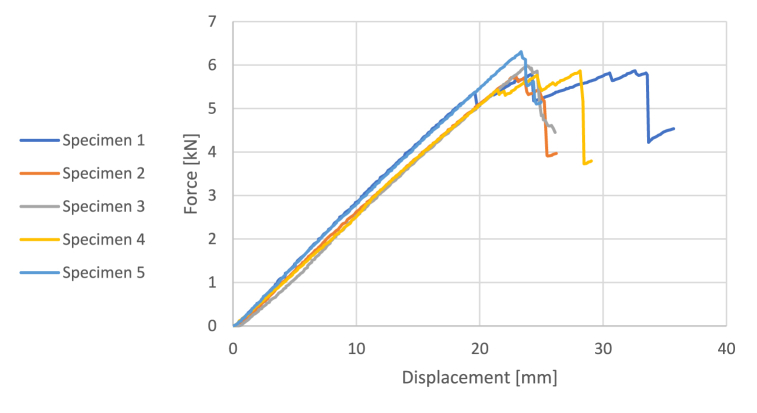
Force-displacement relationship of GFRP.

The tested material is glass fibre-reinforced plastic (GFRP), consisting of an epoxy resin matrix and continuous long glass fibres. The test is carried out on four samples, each with a cross-section of 16 by 40 mm and a span of 45 cm. The obtained force-displacement relationship is shown in Fig. 4.

The maximum force and displacement, the young modulus and the peak stress are shown in the table hereunder. The results are consistent with [16] that gives a range of young modulus between 35000 and 86000 MPa (see Table 1).

### 2.2 3-Point bending test on cellulose ice

**Fig. 5:**
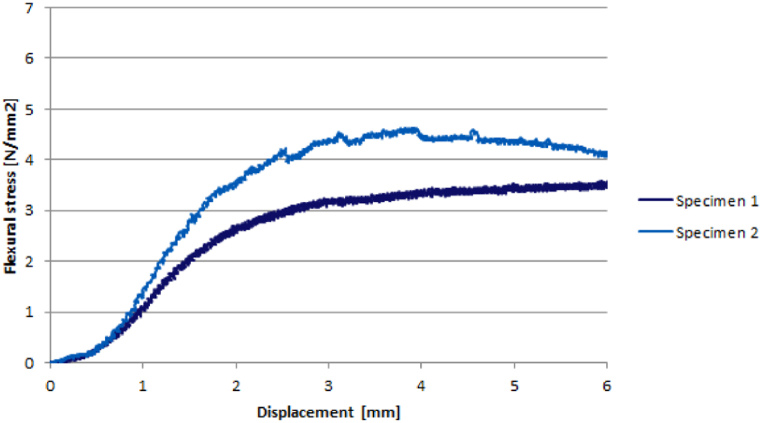
Stress - displacement behaviour of cellulose ice [17].

The bending test was carried out on two samples with a section of 60 by 60 mm and a beam span of 40 cm, as published by Arno et al. [17]. The samples contained 50–75 g of cellulose for every 1000 g of water. The stress-displacement relationship is shown in Fig. 5. Additionally, a compressive test was performed on two samples with dimensions of 100 × 100 × 100 mm. The Young's modulus, peak stress, and compression strength are provided in Table 2.

**Table 1:** 3-Point bending test on GFRP – Results.

Samples	Max Force [kN]	Max displacement [mm]	Elastic modulus [MPa]	Peak stress [MPa]
Specimen 1	−5.87	−32.59	25035	387
Specimen 2	−5.73	−22.95	34698	378
Specimen 3	−5.98	23.84	34923	395
Specimen 4	−5.87	28.14	28985	387
Specimen 5	−6.31	23.35	37559	416
Average	−5.95	3.96	32240	393

**Table 2:** 3-Point bending test on cellullose ice – Results.

Mechanical Proprieties	Elastic modulus [MPa]	Peak stress [MPa]	Compression strength [MPa]
Specimen 1	630	3.85	4.6
Specimen 2	620	4.63	4.6

### 2.3 3-Point bending test on GFRP + cellulose ice

The two materials were tested in a three-point bending test as follows.

- The span of the beam is 45 cm, and the width of the beam is 40 mm.
- The GFRP has a thickness of 16 mm, and the beam has a thickness of 65 mm, resulting in a total height of 81 mm.
- A fabric made of woven cotton is added between the two materials.

**Fig. 6:**
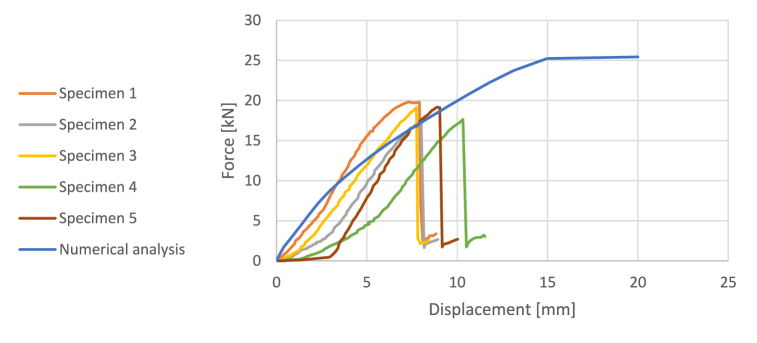
Force-displacement relationship of GFRP + Cellulose ice.

The stress-strain relationship is shown in the Fig. 6. The beam fails by longitudinal shear at the connection as the cellulose ice begins to plasticize. The maximum force, peak stress, and overall Young's modulus are provided in the Table 3.

**Table 3:** 3-Point bending test on GFRP+cellullose ice – Results.

Samples	Maximum force [MPa]	Maximum displacement [mm]	Elastic modulus [MPa]	Peak stress [MPa]
Specimen 1	19.82	7.34	3009	51
Specimen 2	17.62	8.00	2816	45
Specimen 3	19.17	7.75	2829	49
Specimen 4	17.64	10.3	1832	45
Specimen 5	19.12	8.30	2761	49

Compared to the peak stress and Young's modulus of the cellulose ice, the peak stress of the composite beam is significantly increased. Additionally, when compared to the three-point bending tests of the GFRP, the maximum displacements and force are reduced due to the larger cross-section.

### 2.4 Comparison with a numerical analysis

A numerical analysis of the test is performed using ABAQUS FEA software (formerly known as ABAQUS) [18] with the following assumptions.

- The specimen is modelled as a laminated (layered) shell made of two materials.
- The GFRP exhibits elastic brittle behaviour, with a Young's modulus of 32 240 MPa and a maximum stress of 392.6 MPa. These values are the averages from the tests.
- The cellulose ice is characterised by in-plane isotropic behaviour, brittle elastic behaviour in tension and nonlinear ductile behaviour in compression. A Young's modulus of 620 MPa was considered, along with a tensile strength of 0.5 MPa and a compressive strength of 4.6 MPa.
- The supports and load are modelled as two non-deformable rollers.

**Fig. 7:**
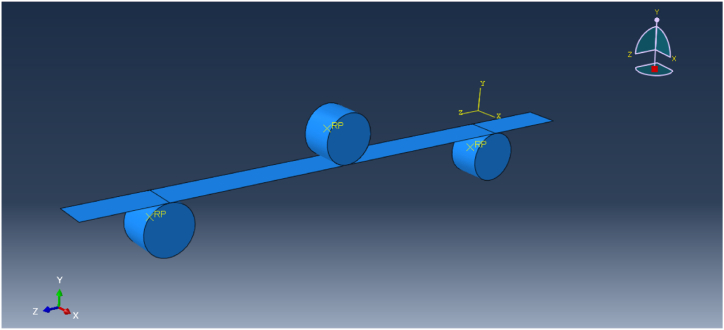
Numerical model in ABAQUS.

The numerical model is shown Fig. 7.

The force-displacement relationship resulting from the numerical simulation is shown in Fig. 6, along with a comparison to the experimental tests. The model captures the behaviour well during the initial elastic phase. Some discrepancies are attributed to the variability of the cellulose ice and the numerical model's exclusion of the initial setup phase, where the setup contacts the specimen and the materials interact. Additionally, the elasticity of the connection is not accounted for in the model.

As the tension in the ice reaches its maximum tensile stress, the slope of the numerical model decreases. The maximum tensile stress of the specimen is not precisely known, and a conservative value of 0.5 MPa was used.

The failure mode is not accurately captured because the model does not consider the failure of the connection. Instead, the numerical model indicates failure due to the plasticization of the cellulose ice.

### 2.5 Discussion

The action of the two materials is well defined.

- FRP enhances the resistance of the section due to its excellent tensile properties.
- Cellulose ice increases the massiveness of the section and contributes to a more ductile behaviour.

However, the elasticity of the connection between FRP and cellulose ice is an important parameter and not considered in the numerical model. As a result, the model fails to accurately capture the behaviour beyond the elastic phase of the materials and cannot reproduce the failure mode. This limitation arises from the assumption of a perfect connection and material failure preceding any connection failure. Therefore, the model is valid only for a low range of stresses, particularly in the elastic phase. This numerical model is applied in the context of a monumental sculpture, considering these limitations.

## 3 Application to the structural behaviour of a monumental sculpture

This section details how the previously discussed principles were applied to a monumental sculpture, covering the design, hypotheses, and results.

### 3.1 Design of the sculpture and structural components

**Fig. 8:**
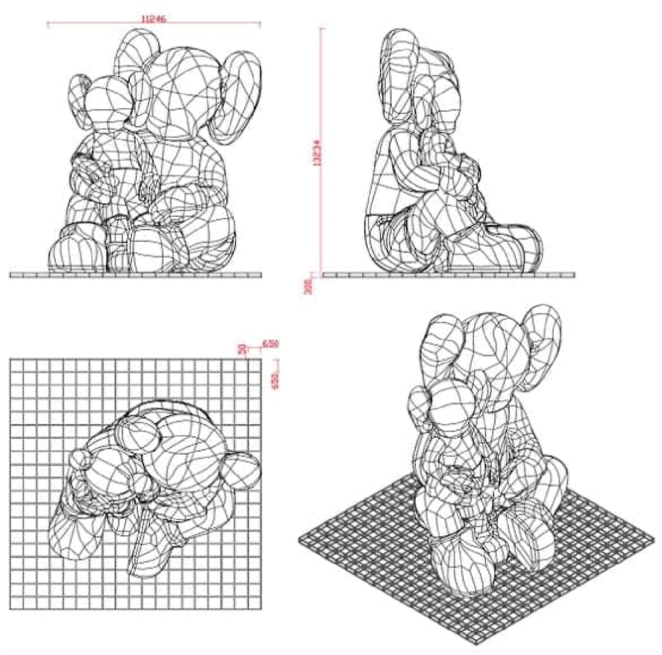
Initial design of the monumental sculpture.

The sculpture, created by the American artist and designer Brian Donnelly, who is professionally known as Kaws (often styled as KAWS), exhibits his signature style. KAWS is renowned for his reinterpretations of existing cultural icons. This particular sculpture features two imposing “Companion” characters. Drawing inspiration from Mickey Mouse, these characters are distinctively marked by two bones protruding from their heads. The larger figure of the two figures stands at a height of 13 m, as illustrated in Fig. 8.

**Fig. 9:**
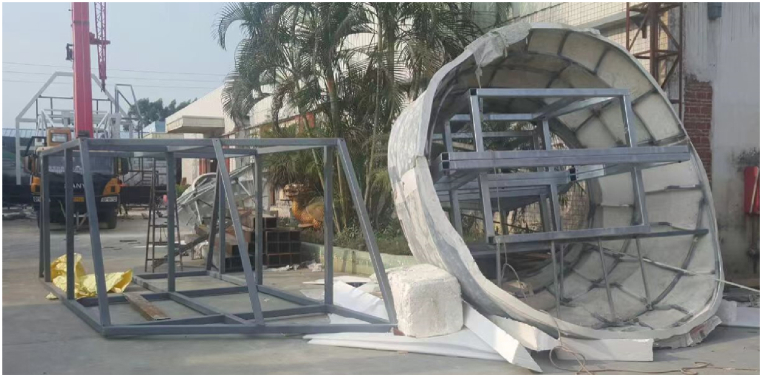
Steel structure with FRP covered with fabric.

The sculpture incorporates an internal steel framework, which is essential for supporting the Fiber-Reinforced Polymer (FRP) moulds. Fig. 9 illustrates how the steel structure support the FRP components.

### 3.2 ABAQUS model

**Fig. 10:**
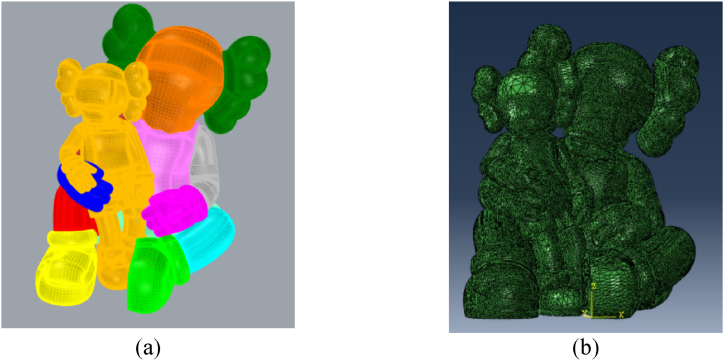
Model of the characters: (a) in Rhino (b) in ABAQUS®.

**Fig. 11:**
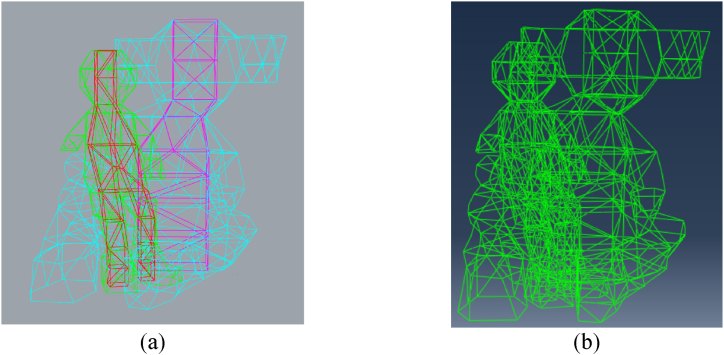
Model of the frame: (a) in Rhino (b) in ABAQUS®.

The model was created using a precise 3D representation of the characters, later refined in GiD® for compatibility with ABAQUS FEA (as shown in Fig. 10). The mesh was generated using three-dimensional triangular shell elements with reduced integration. Simultaneously, the steel framework was modelled in Rhino and converted into a mesh using GiD® (as depicted in Fig. 11). In this frame, two-node linear beam elements were used, with rigid connections between them as the steel elements are hollow square section welded together. The dimensioning of the steel structure is not the focus of this article. It was the subject of a separate study by the project engineers based on the recommendations given after the study of this model. Two simulations are carried out: one considering only the FRP and one considering the action of cellulose ice.

### 3.3 Material proprieties

This section presents the material properties implemented in ABAQUS for the sculpture, which comprises three parts, each made of a different material.

- A layer of 3 mm of GFRP.
- A layer of 5 cm ice-based material (the thickness is discussed in section 3.7).
- A steel structure.

**Fig. 12:**
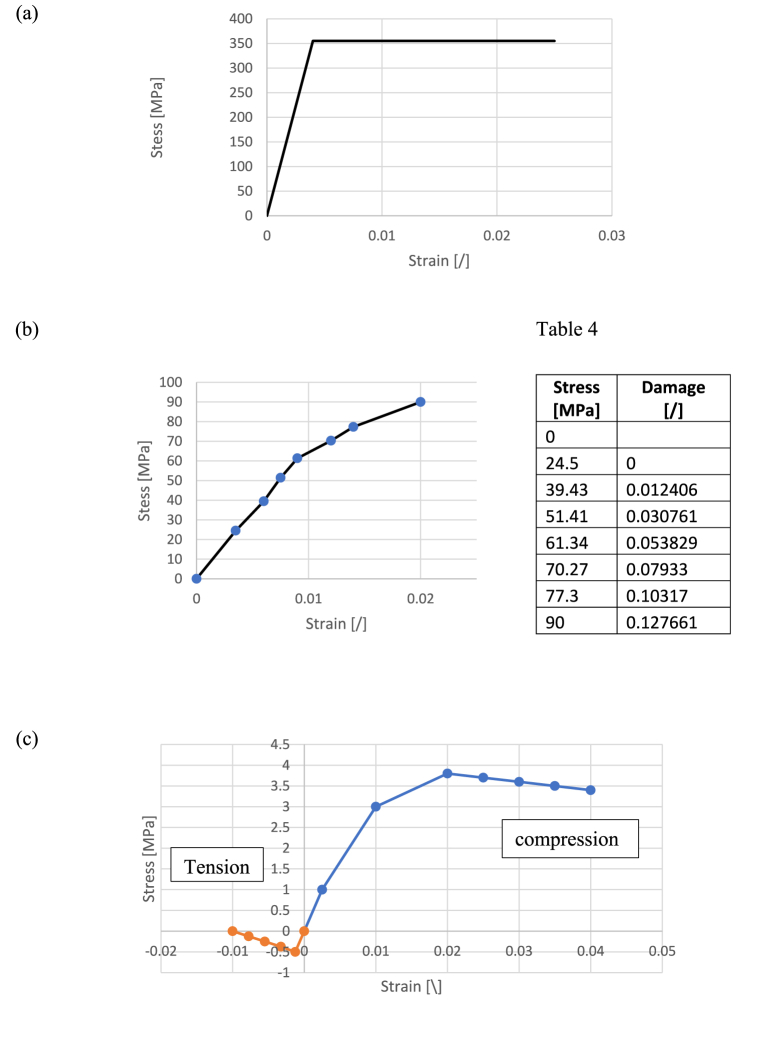
Stress-strain relationship of the materials: (a) Steel, (b)GFRP (c)Ice-based material.

**Fig. 13:**
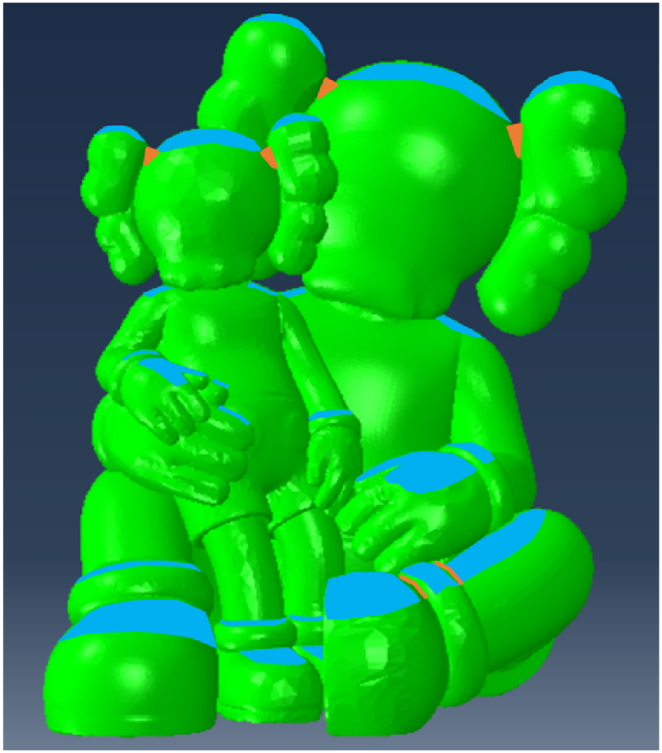
Snow load.

Fig. 12 shows the stress-strain relationship for each material, while Table 5 provides the main mechanical properties (see also Fig. 13).

#### 3.3.1 Steel

The steel grade is S355 and usual mechanical properties are considered. Steel is modelled as an elastic perfectly plastic material (Fig. 12 (a)).

#### 3.3.2 GFRP

The mechanical properties of reinforced plastics vary widely depending on factors such as the fibre-to-matrix ratio, manufacturing techniques, mechanical characteristics of constituent materials, and fibre orientation within the matrix [[16], [19], [20], [21]].

The specific FRP used for the project differs from the materiel tested in section 2.1. It is a Glass Fibre-Reinforced Polymer (GFRP) with randomly oriented short glass fibre reinforced composites, manufactured using hand lay-up moulding. This process involves manually placing glass fibre reinforcements in a mould and applying polyester resin with a roller to remove trapped air [22]. In the sculpture, the end product contains approximately 25–30 % fibre by weight. This material is usually characterized by damageable-elastic behaviour in tension and compression.

The mechanical behaviour that is considered is based on the [23] that presents the results of an experimental programme carried out to characterise the mechanical behaviour of random short glass fiber reinforced composites. In tension, the average elastic modulus, and tensile strength is 7.08 GPa and 91.6 MPa. The material initially exhibits linear behaviour and then nonlinear behaviour once stress reaches approximately 30 MPa. The compressive modulus is found to be equal to 8.33 GPa and the compressive strength is 153 MPa.

In the numerical simulation, symmetrical in-plane isotropic behaviour and damageable-elastic behaviour in tension and compression are considered. For simplicity symmetrical behaviour is assumed with elastic modulus and strength equal to 7.08 GPa and 91.6 MPa. Damage variables are defined as in Ref. [24]. The stress-strain relationship considered is shown on (Fig. 12 (b)). The damage variable are provided in the Table 4.

**Table 4:** Damage variable of GFRP.

Stress [MPa]	Damage [/]
0	
24.5	0
39.43	0.012406
51.41	0.030761
61.34	0.053829
70.27	0.07933
77.3	0.10317
90	0.127661

**Table 5:** Mechanical Proprieties of the materials.

Mechanical Proprieties	Tensile strength [MPa]	Compressive strength [MPa]	Elastic modulus [MPa]	Density [kg/m³]
Steel	355	−355	210 000	7580
Glass(fibre) Reinforced Plastic	91.6	−91.6	7080	1350
Cellulose ice	0.5	−3.8	420	900

#### 3.3.3 Ice-based material

Ice-based materials are characterised by in-plane isotropic behaviour, brittle elastic behaviour in tension, and nonlinear behaviour in compression. In this paper, the ice-based material includes a mixture ratio of 2% pulp fibre board, similar to the specimens tested in Ref. [25].

A statistical model developed in this research is used to determine the mechanical properties in compression. This model is based on uniaxial compressive tests under 20 different cases, investigating the effects of temperature (−20°C, −15°C, −10°C, −5°C) and fibre content (0%, 1%, 2%, 4%, 6%) on compression. Young modulus, peak stress, and strain are evaluated as follows [25]:

Equation 1 $$\begin{array}{c} \frac{f_{c}}{f_{0}}=0.14-0.2\times \frac{T}{T_{0}}+1.83{\left(\frac{\omega }{\omega_{0}}\right)}^{0.56}\\ f_{c}=\left(0.14-0.2\times \frac{\left(-5\right)}{1}+1.83{\left(\frac{2}{1}\right)}^{0.56}\right)\times 1MPa=3.8MPa \end{array}$$

Equation 2 $$\begin{array}{c} \frac{\varepsilon_{c}}{\varepsilon_{0}}=1.11-0.032\times \frac{T}{T_{0}}+1.107\frac{\omega }{\omega_{0}}\\ \varepsilon_{c}=\left(1.11-0.032\times \frac{\left(-5\right)}{1}+1.1071\right)\times 0.01=0.014 \end{array}$$

Equation 3 $$\begin{array}{c} \frac{E_{c}}{E_{0}}=0.45-0.019\times \frac{T}{T_{0}}+0.087{\left(\frac{\omega }{\omega_{0}}\right)}^{0.54}\\ E_{c}=\left(0.45-0.019\times \frac{\left(-5\right)}{1}+0.087{\left(\frac{2}{1}\right)}^{0.54}\right)\times 1GPa=0.42 \end{array}$$

The model is completed with additional experimental data. The final stress-strain relationship is shown in Fig. 12 (b).

In tension, brittle elastic behaviour is considered with a maximum stress of 0.5 MPa.

### 3.4 Loads

For the design of the characters and the steel structure, the considered loads include.

• Self-weight of FRP and steel structure
• Dead load of the ice composite
• Snow load
• Wind load

#### 3.4.1 Self-weight and dead load

The gravitational acceleration is considered to be [9.81 m/s^2^]. The self-weight of FRP and steel structure and the dead load of the ice is calculated in Table 5.

#### 3.4.2 Snow load

In accordance with the GB50009-2012 Load Code for the Design of Building Structures [26], the characteristic snow load value is calculates as follows:

Equation 4 $$S_{k}=\mu_{r}s_{0}$$

$\mu_{r}$ is the distribution factor for roof snow load and $s_{0}$ is the reference pressure.

The reference snow pressure is adopted with a 50-year recurrence. The value is found in the Table E.5 of Appendix E [26] for the Changbai Mountain and is equal to 0.6 kN/m^2^. The distribution factor for roof snow is determined using Table 7.2.1 [26]. The snow load is applied to specific areas, as illustrated in Fig. 13. Blue zones follow item 3 of Table 7.2.1 [26], while orange zones indicate areas of snow accumulation. A non-uniform distribution of the factor $\mu_{r}$ is considered in the orange zones, adhering to item 6 (non-uniform distribution 2).

#### 3.4.3 Wind load

The wind load is calculated as follows using the formula:

Equation 5 $$w_{k}=\beta_{z}\mu_{s}\mu_{z}w_{0}$$

**Fig. 14:**
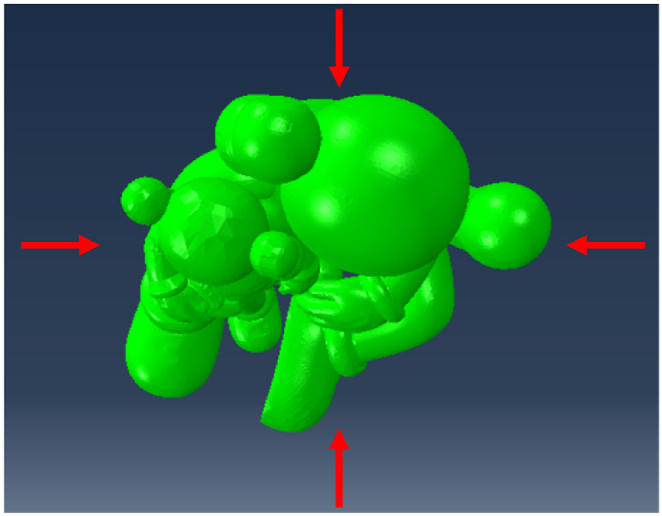
Wind load.

Here, the dynamic response factor $\beta_{z}$ is set to 1, the exposure factor $\mu_{z}$ is 2.91 (corresponding to Terrain roughness category B), and $w_{0}$ is 0.45 kN/m^2^ (refer to Table E.5 in Appendix E [26]). Given the complex shape of the sculpture, determining the shape factor $\mu_{s}$ involves simplifying the sculpture to a cylindrical tower (item 37 in Table 8.3.1 [26]). The tower has a diameter of 10 m and a height of 13 m. Four wind directions were considered, as illustrated in Fig. 14.

### 3.5 Combinations

In accordance with Chinese Standards [26], the following Ultimate Limit State (ULS) combinations are considered, involving different types of loads - D for dead load, S for snow load, and W for wind load:

Equation 6 $$\begin{array}{c} \left(1\right)\;1.35D+0.98S+0.84W\\ \left(2\right)\;1.2D+1.4W+0.98S\\ \left(3\right)\;1.2D+1.4S+0.84W \end{array}$$

### 3.6 Frontier conditions

**Fig. 15:**
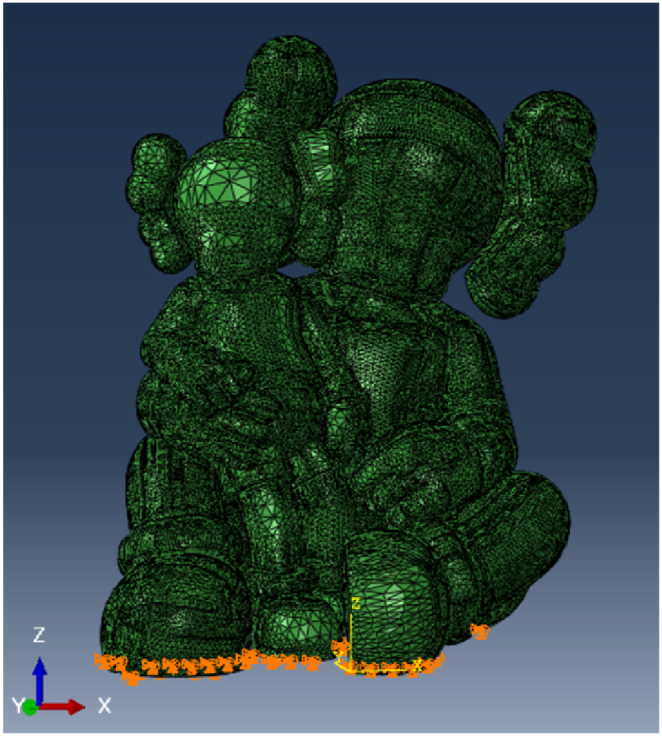
Frontier conditions.

Given that the sculpture is positioned with minimal fixings on a plate firmly anchored in the ground, it is assumed that only translational movements are restricted (Fig. 15).

### 3.7 Determination of the thickness of ice

**Fig. 16:**
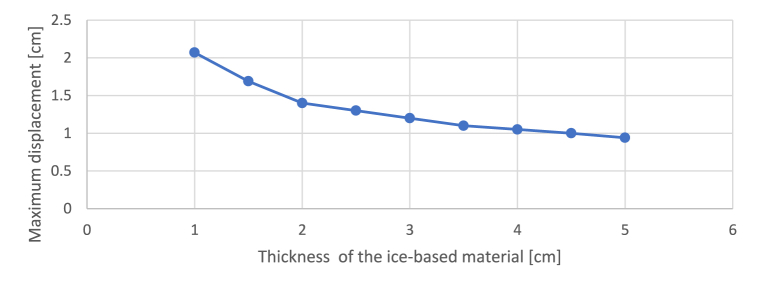
Determination of the thickness of ice based on the maximum displacement.

The thickness of the GFRP was set at 3 mm due to technical constraints. This thickness was determined through iteration to minimise the movement of the hand of the large sculpture, which could not be adequately supported by the steel structure. Values ranging from 1 to 5 cm were tested, and the results are shown in the Fig. 16.

### 3.8 Results

The results are detailed with displacements quantified in metres [m] and stresses expressed in Newtons per square metre [N/m^2^], which is equivalent to 1E-6 Megapascals [MPa]. Among the various scenarios examined, the most critical is identified as the “front wind” condition. This particular scenario involves the sculpture being subjected to wind forces from the y-direction, in particular under the load combination designated as (2).

The mesh has a size of 10 cm on average, except on certain areas such as the fronts of the sculptures or the shoes where the mesh is larger. In sensitive parts, the mesh is locally refined to 1 cm (such as connections between parts of the body).

#### 3.8.1 Displacements

**Fig. 17:**
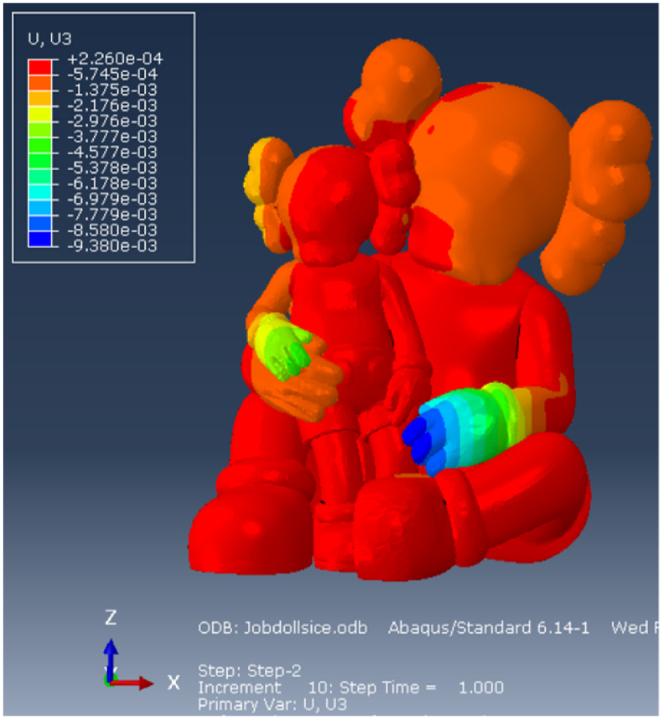
Vertical displacements [m]: FRP + Cellulose Ice.

**Fig. 18:**
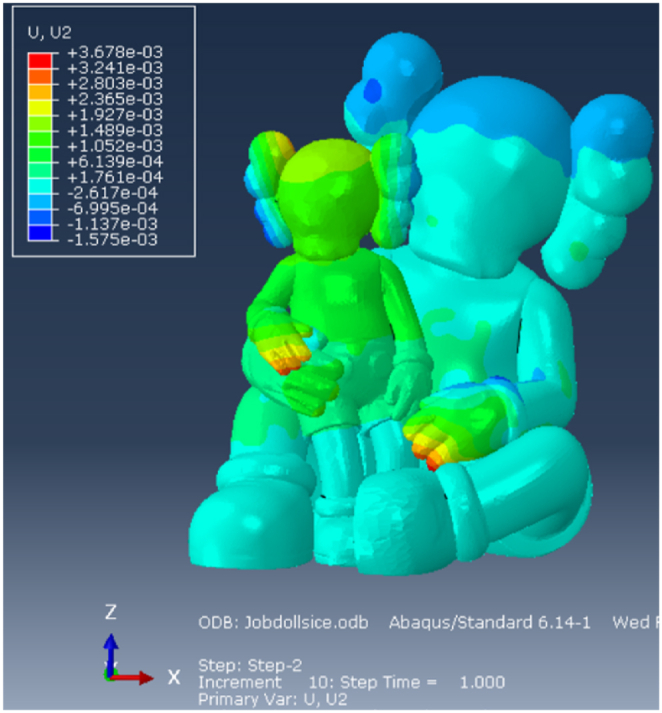
Horizontal displacements [m]: FRP + Cellulose Ice.

The vertical displacements are illustrated in Fig. 17. The largest vertical displacement in occurs at the hand of the larger character, measuring 9.4 mm that is primarily attributed to the absence of a supporting steel structure. It is indeed very difficult to place one there. The vertical displacements in the intertwined hands and the ears of the smaller character are also notably higher compared to other parts of the sculpture. The horizontal displacement, shown in Fig. 18, are limited to less than 1 cm.

#### 3.8.2 Stresses

**Fig. 19:**
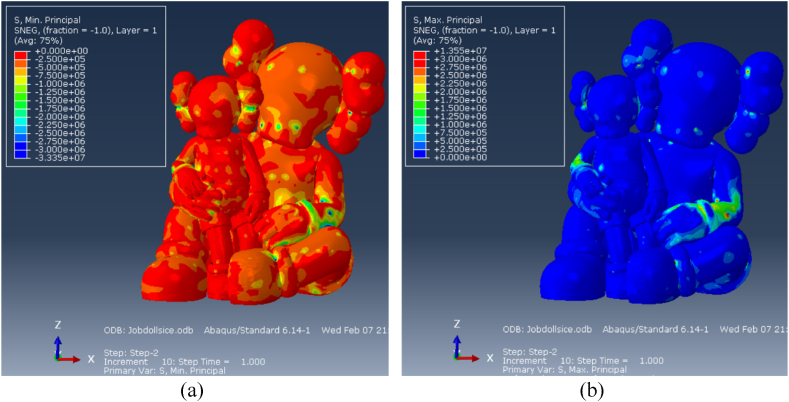
Layer GFRP [N/m^2^]: (a) Maximum principal stress, (b) Minimum principal stress.

**Fig. 20:**
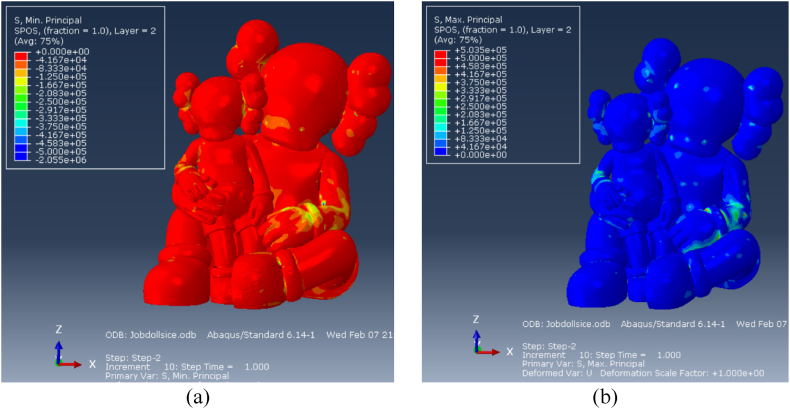
Layer ice-based material [N/m^2^]: (a) Maximum principal stress, (b) Minimum principal stress.

The compressive stress are depicted in Fig. 19, Fig. 20.

For the FRP layer, principal stresses predominantly stay under 30 MPa (in absolute value), ensuring the material remains within its elastic phase (Fig. 19).

In Fig. 20 (a), the principal compressive stresses of the ice-based material layer is under 3 MPa in absolute value, meaning that the material stayed in the elastic range. The maximum principal stresses (Fig. 20 (b)) are generally below 0.5 MPa. There are however still significant stress around the hand of the larger character, indicating a potential risk of loss of cohesion and failure in this area. For this reason, the hand of the larger character was reinforced with steel rebars.

## 4 Execution

**Fig. 21:**
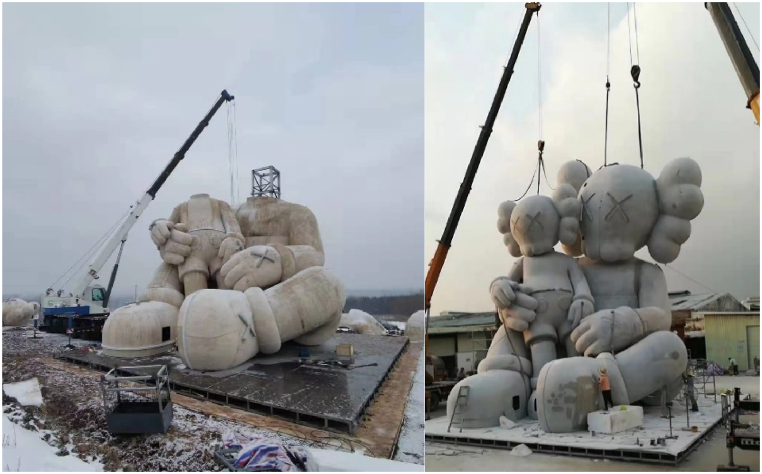
Assembly of steel frame and FRP.

**Fig. 22:**
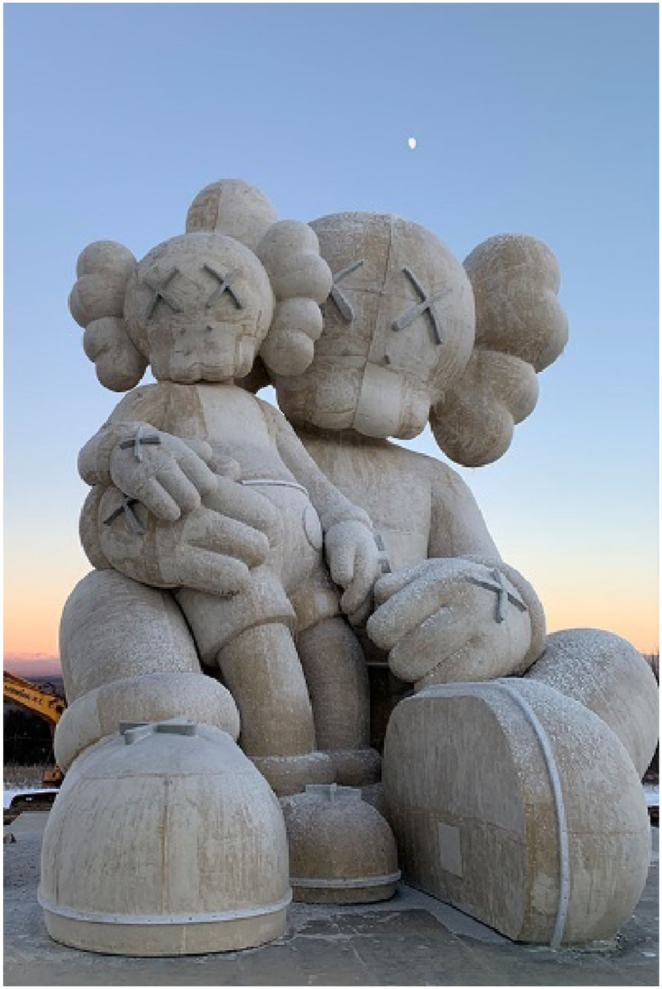
FRP covered with fabric.

**Fig. 23:**
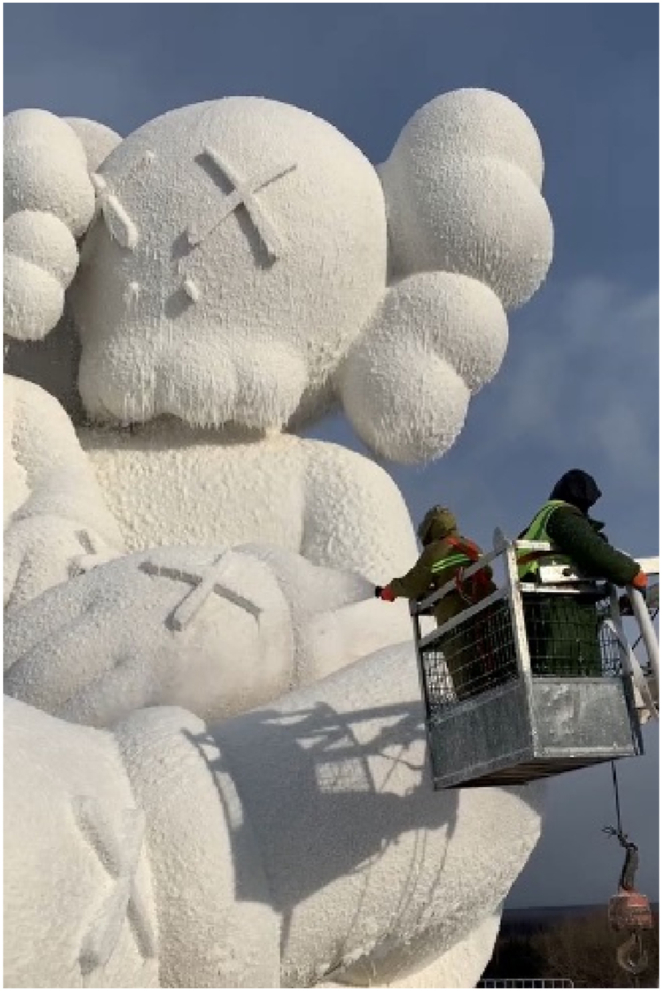
Spraying.

**Fig. 24:**
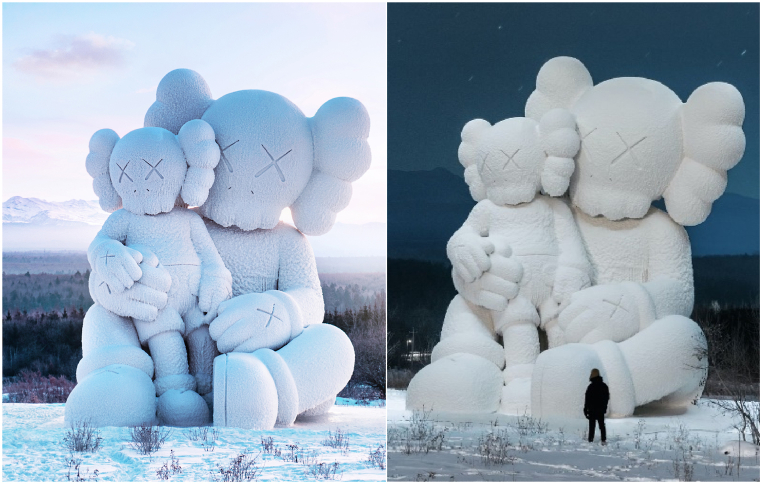
Final result of ice sculpture.

Constructed in Changbai Mountains near the North Korean border at an elevation of 2750 m, the sculpture faced environmental challenges during its creation. Usually, the area endures harsh winters with temperatures plummeting to −30 °C. However, this year deviated from the norm, presenting unusually warm temperatures. This unexpected warmth led to snowmelt and soil softening and complicated the construction process. To counter these problematic conditions, the sculpture was placed on a platform designed to evenly distribute its weight across the softened terrain, as shown in Fig. 21. The construction process involved the synchronized assembly of the steel framework and GFRP forms. This task was managed with the aid of cranes, as depicted in Fig. 21. Following the assembly of the steel structure and forms (Fig. 22), the next phase involved applying a water-cellulose mixture onto the FRP structure. The process is illustrated in Fig. 23 and resulted in the final sculpture showed (see Fig. 24).

## 5 Conclusion

This paper has explored the combination of ice-based materials with FRP to improve bending capacity through three-point bending tests. By analysing the mechanical behaviour of composite materials compared to individual components, insights were gained into the potential of this material combination. However, limitations in numerical modelling, particularly regarding connection elasticity and failure modes, underscore the need for further research and refinement in this area.

The application of these principles to the structural behaviour of a monumental sculpture, exemplified by the design and construction process of a sculpture by contemporary artist KAWS, demonstrates the practical implications of this research. Through precise modelling and analysis, coupled with innovative construction techniques, ice-based materials and FRP offer exciting possibilities for the creation of architecturally significant structures in challenging environments.

## CRediT authorship contribution statement

**Arno Pronk:** Supervision, Conceptualization. **Elke Mergny:** Writing – original draft, Validation, Software, Methodology, Conceptualization. **Qingpeng Li:** Validation, Supervision, Methodology, Conceptualization. **Yiling Zhou:** Conceptualization.

## Declaration of generative AI and AI-assisted technologies in the writing process

During the preparation of this work the author(s) used CHATGPT in order to improve some sentences in the abstract, introduction and conclusion. After using CHATGPT, the authors reviewed and edited the content as needed and take full responsibility for the content of the publication.

## Declaration of competing interest

The authors declare that they have no known competing financial interests or personal relationships that could have appeared to influence the work reported in this paper.
